# Facts and prospects of peptide in targeted therapy and immune regulation against triple-negative breast cancer

**DOI:** 10.3389/fimmu.2023.1255820

**Published:** 2023-08-25

**Authors:** Yongxiu Huang, Anqi Zeng, Linjiang Song

**Affiliations:** ^1^ School of Medical and Life Sciences, Chengdu University of Traditional Chinese Medicine, Chengdu, China; ^2^ Institute of Translational Pharmacology and Clinical Application, Sichuan Academy of Chinese Medical Science, Chengdu, Sichuan, China

**Keywords:** triple-negative breast cancer, peptide, immunity, target, combination

## Abstract

Triple-negative breast cancer (TNBC) is the most aggressive subtype of breast cancer. Due to the lack of specific therapeutic targets, treatment options are limited, and the recurrence and metastasis rate is high, the overall survival of patients is poor. However, with the discovery of some new targets and the corresponding immune regulation after targeting these targets, TNBC has a new hope in treatment. The peptide has a simple structure, strong binding affinity, and high stability, and has great potential in targeted therapy and immune regulation against TNBC. This review will discuss how single peptides and peptide combinations target triple-negative breast cancer to exert immunomodulatory effects. Among them, single peptides target specific receptors on TNBC cells, act as decoys to target key ligands in the regulatory pathway, and target TME-related cells. The combinations of peptides work in the form of cancer vaccines, engineered exosomes, microRNAs and other immune-related molecular pathways, immune checkpoint inhibitors, chimeric antigen receptor T cells, and drug-peptide conjugates. This article is mainly dedicated to exploring new treatment methods for TNBC to improve the curative effect and prolong the survival time of patients.

## 1 Introduction

Breast cancer is one of the most frequently diagnosed cancers among all cancers and the most common cancer among women worldwide (1), posing a serious threat to the health of women. Breast cancer is a highly heterogeneous malignant tumor. Breast cancer cell lines are usually used as the original model to study it. However, because the cell line is cultured *in vitro* alone, it lacks the influence of the microenvironment and cannot fully simulate the heterogeneity of cancer cells at the real tumor level. It is also necessary to combine tumor samples or mouse models to verify the results (2). In addition, breast cancer can be divided into different subtypes, but often because of different classification criteria and seem to be somewhat confused, so after considering the genetic and epigenetic aspects of the tumor, it can be divided into lumen A, lumen B, HER2 positive, triple negative A and triple-negative B five subtypes(2). The luminal A and B cell lines are distinguished from each other, and the HER2-positive line is identified as a single subtype, while triple-negative breast cancer (TNBC) is due to the absence of estrogen receptor (ER), progesterone receptor (PR) and human epidermal growth factor receptor 2 (HER2)(3). In addition, TNBC contains at least four subcategories, namely, metaplastic breast cancer, core basal carcinoma, low-density protein, and interferon-rich breast cancer, but there are only two subgroups of TNBC cell lines currently available, namely triple-negative A and B cell lines. Among them, triple-negative A is mainly characterized by the expression of basal keratin (KRT4/5/6/13/14/15/16/17), similar to the core basal tumor, while triple-negative B is characterized by cancer stem cell patterns such as CD44+, CD24- and migration markers such as VIM(2). It is hoped that more TNBC cell lines, such as interferon-rich cell lines, can be established in the future to cover the current subclass of TNBC more comprehensively.

TNBC accounts for about 10% to 20% of all types of breast cancer and is more common in young women of African and Hispanic origin (4). However, the incidence of TNBC in China has also increased year by year since 2000. Therefore, TNBC also poses new challenges to the treatment of breast cancer. Due to the lack of iconic ER, PR, and HER2 receptors in TNBC, endocrine therapy and targeted therapy commonly used in breast cancer treatment cannot be used (5). At present, the treatment of TNBC includes surgery, adjuvant chemotherapy, and adjuvant radiotherapy (6). Usually, surgical resection of the tumor mass is combined with a variety of drug treatments such as chemotherapy, radiotherapy, and immunotherapy (7). Chemotherapy is the main treatment (8). The commonly used chemotherapeutic drugs include anthracyclines, taxanes, and cyclophosphamide (9). Although TNBC is sensitive to chemotherapy, chemotherapy drugs lack specificity and often damage healthy tissues. Toxic side effects often occur during treatment, such as neurotoxicity and nephrotoxicity (10). With the resistance of tumor cells to chemotherapeutic drugs, it is gradually unable to control the proliferation of tumor cells (11). And chemotherapy drugs have poor pharmacokinetics and rapid systemic clearance caused by poor water solubility (12). Due to these reasons, only 20% of TNBC patients showed pathological complete response (PCR) to neoadjuvant chemotherapy (13), and many patients experienced treatment failure. Although adjuvant radiotherapy using radiotherapy and imaging technology has a certain therapeutic effect on TNBC, systemic reactions caused by off-target toxicity and radiation resistance of tumor cells limit the effectiveness of clinical treatment(14). In addition, it has the characteristics of rapid progression and strong invasiveness (5). TNBC patients often have distant brain, liver, lung, and bone metastasis, and poor prognosis (15). Therefore, TNBC is still the most difficult subtype of breast cancer (16). Although the overall 5-year survival rate of breast cancer is 90%, the 5-year mortality rate of TNBC is 77% regardless of stage, and the median death time of patients is 4.2 years (17).

In the face of the lack of effective treatment for TNBC, it is particularly critical to find new treatment methods (18). One of the ideas is to explore new molecular targets for targeted therapy and immune regulation to control the growth and metastasis of tumor cells, reduce targeted toxicity and prevent recurrence (19). The reason why there is currently no targeted therapy for TNBC is closely related to the fact that TNBC is a heterogeneous tumor (20). Tumor heterogeneity refers to the changes in the genetic or epigenetic evolution of tumor cells during clonal evolution (19). Despite this, there are still overexpressed targets in TNBC that can be used for specific targeted therapy (14). Now there are targeted therapy methods under study such as immune checkpoint inhibitors, cell DNA key repair enzyme inhibitors (21), vascular endothelial growth factor inhibitors, and integrin inhibitors (22). These new molecular targets are up-regulated receptors such as folate receptors and integrin receptors in TNBC. The development of new therapies for new molecular targets in the treatment of TNBC tumors is becoming a research hotspot (23).

**Figure 1:**
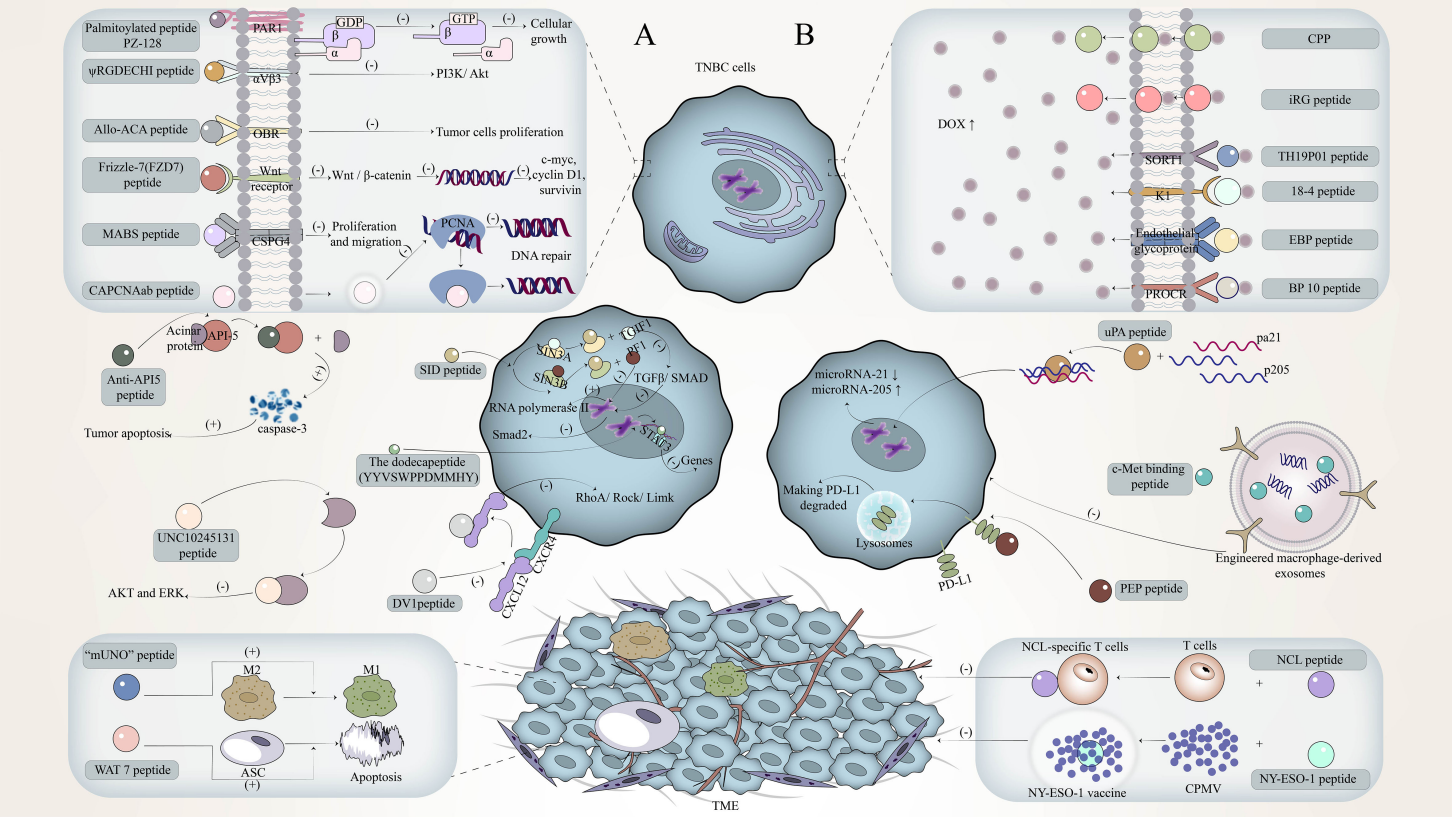
The mechanism of peptide in targeted therapy and immune regulation against triple-negative breast cancer (TNBC). **(A)** The mechanism by which single peptides target TNBC to exert its immunomodulatory effects. **(B)** The mechanism by which the combinations of peptides target TNBC to exert its immunomodulatory effects.

Another problem to be solved after finding new molecular targets is to develop active targeting ligands, and peptides are a good candidate (24). Peptides have a series of advantages such as simple structure, low synthesis cost, easy engineering (25), strong binding affinity, and high stability(26), which can efficiently reach the target and have great potential in the treatment of TNBC. Many targeted peptides have been found in phage display and *in vivo* biopanning techniques and can be further engineered (25). These peptides can achieve immune regulation and anti-tumor by targeting the corresponding molecular targets in TNBC, and these peptides can work alone or in combination with other substances such as adjuvants. Among them, a single peptide plays a role mainly by targeting specific receptors on TNBC tumor cells and inhibiting the growth, proliferation, and metastasis of tumor cells through certain molecular mechanisms; or as an inducing peptide targeting a key ligand in the regulatory pathway of triple-negative breast cancer to promote tumor cell death; there is also a peptide single peptide targeting triple-negative breast cancer-related cells to play an immunomodulatory role. The combinations of peptides work by forming cancer vaccines, engineered exosomes, microRNAs and other immune-related molecular pathways, immune checkpoint inhibitors (ICI), chimeric antigen receptor T cells, and drug-peptide conjugates to improve efficacy **Figure 1**. It can be seen that peptides have great hope in targeted therapy and immune regulation of TNBC.

Although peptides are promising for the treatment of TNBC in the future, current research has focused on reporting the discovery of new therapeutic molecular targets or potential therapeutic molecular targets, and there are few reports summarizing peptides for targeted therapy and immune regulation of TNBC. Therefore, this article will focus on how the peptide is targeted therapy and immune regulation to achieve anti-TNBC, which will provide a reference for exploring new treatment methods for TNBC, enhancing the clinical treatment effect of TNBC, and improving the survival rate of TNBC patients.

## 2 Single peptides target TNBC to exert immunomodulatory effects

Single peptides are often widely studied because of their simple structure and ease to explore their independent mechanism of action (27). For how a single peptides target TNBC and play an immunomodulatory role, we will discuss it in three parts: targeting specific receptors on TNBC cells, as decoys targeting key ligands in the regulatory pathway, and targeting TME-related cells **Table 1**.

**Table 1:** The mechanism by which single peptides target TNBC to exert its immunomodulatory effects.

Catalog		Peptide name	Peptide sequence	Target	Mechanism	Reference
Specific receptors on triple-negative breast cancer cells	G protein-coupled receptors	LRH7-G5	/	GPR1	Inhibiting the Chemerin/ GPR1 signaling	(3)
		palmitoylated peptide PZ-128	AEEQNPWARYLEWLFPTELLLELC	PAR1	Inhibiting the PAR1/ G protein signaling	(5)
		cytotoxic cyclic peptide SA-1	/	ADORA2B	Inhibiting the expression of ADORA2B	(28)
		CP-B1Ras	/	B1R	Inhibiting the phosphorylation of MAPK p42/ p44	(29)
		CP-B2Ras	/	B2R	Activating the p38kinase/ p27kip1 pathway Inhibiting the growth of TNBC in the G1 phase	(18)
	Integrin receptor	a type IV collagen-derived peptide AXT050	/	αVβ3	Inhibiting the formation of new blood vessels	(30)
		ψRGDECHI	/	αVβ3	Inhibiting the PI3K/ Akt pathway	(31)
	Leptin receptor	allo-ACA	H-alloThr-Glu-Nva-Val-Ala-Leu-Ser-Arg- Aca-NH2	OBR	Inhibiting leptin-stimulated tumor cell proliferation Anti-angiogenic therapy	(32)
	Chondroitin sulfate receptor	MABS	/	CSPG4	Inhibiting the proliferation and migration of TNBC	(33)
	Wnt receptor	Frizzle-7(FZD7)	/	Wnt receptor	Inhibiting the Wnt / β-catenin signaling pathway Inhibiting c-myc, cyclin D1, survivin and other target genes Inhibiting tumor angiogenesis	(34)
	Proliferating cell nuclear antigen	CAPCNAab	LGIPEQEYSC	CAPCNA	Inhibiting the repair of DNA	(35)
Peptide decoys	Chemotactic factor	DV1peptide	L-G-A-S-W-H-R-P-D-K-C-C-L-G-Y-Q-K-R-P-L-P-A (β-azido)- CONH2	CXCL12	Inhibiting CXCR4-CXCL12 Inhibiting the RhoA/ Rock/ Limk signaling pathway Inhibiting Akt	(36)
	SIN3	SID peptide	/	SIN3A SIN3B	Inhibiting the Wnt / β-catenin Inhibiting the Axin2 and Bcl9 Inhibiting the TGFβ/ SMAD signal transduction pathway Inhibiting the EMT	(37)
	Transcription factor	ASRPS	MTTKMRRLRPSAPSGLGQEQEAEVVEGCFPAVTETPFAPAYIKKRGGRIWSSDGEH	STAT3	Inhibiting the STAT3 vascular endothelial growth factor (VEGF) signaling pathway	(38)
		the dodecapeptide	YYVSWPPDMMHY	STAT3	Inhibiting genes related to angiogenesis (VECGF-A), proliferation-related genes and invasion-related genes (MMP1 and MMP7)	(39)
	Anti-apoptotic	anti-API5 peptide	RQIKIWFQNRMKWKKAK LNAEKLKDFKIRLQYFARGLQVYIRQLRALQGKT	API-5	Inhibiting the API-5/ acinar interaction Activating the caspase-3 pathway	(40)
	CIB1	UNC10245131	YKQPYWLINWCS	CIB1	Inhibiting the regulation of AKT and ERK carcinogenic pathways	(41)
		UNC10245092	NH3-EDGGSFWYGAMKALYG	CIB1	Inhibiting the regulation of AKT and ERK carcinogenic pathways	(1)
TME-related cells	Tumor-infiltrating immune cell	the melittin KLA8-26	VLTTGLPALISWIKRKRQQGGGGS-d[KLAKLAKKLAKLAK]	M2	Inducing M2-like macrophagesl death	(42)
		a cyclic peptide	CSSTRESAC	vitamin D receptor on the cell surface of M2	Inducing M2-like macrophagesl death	(6)
		“mUNO” peptide	/	CD 206-positive M2	Converting M2 into M1	(43)
		C24 D peptide	/	Tumor-associated immune cells with CD45 receptor	Re-activating the Src family of tyrosine kinases Activating 69+T cells and 69+ NK cells Activating CD8+ and CD56+ tumor-infiltrating cells	(44)
	Tumor-associated fibroblasts and adipose stromal cells	WAT 7	CSWKYWFGEC	ASC	Inducing the apoptosis of ASC	(45)

/, The structure of the peptide is not mentioned in the current reference, and it is not clear.

### 2.1 Single peptides target specific receptors on TNBC cells

TNBC cells were once unable to target therapy due to the lack of specific receptors, but recent studies have found some potential targets or become the key to targeting triple-negative breast cancer. Using a single peptide to target these receptors has a good anti-TNBC effect.

#### 2.1.1 G protein-coupled receptors

G protein-coupled receptors (GPCRs) are cell surface proteins that are closely related to the occurrence and development of tumors (29). They are involved in tumor growth, angiogenesis, invasion, and metastasis (46), and the expression of GPR1 in TNBC tissues will increase (3). Therefore, GPCRs have also become a good therapeutic target.

Studies have found that an adipokine Chemerin secreted by white adipose tissue can activate GPR1 to release intracellular calcium, thereby inhibiting the accumulation of cyclic adenosine monophosphate, achieving phosphorylation of P42-P44 MAP kinase through G1 heterotrimeric G protein(47). The corresponding peptide antagonist LRH7-G5 can competitively bind to GPR1 with Chemerin, thereby blocking the signal transduction of Chemerin/GPR1 through the P13K/Akt signaling pathway and inhibiting the growth of TNBC cells (3). In addition, protease-activated receptor 1 (PAR1) is also a G protein-coupled receptor. A palmitoylated peptide PZ-128, which targets the intracellular loop of PAR1, can inhibit PAR1/G protein signaling by interacting with GPCRs to significantly inhibit the growth of MDA-MB-231 TNBC cells (5). In addition to the G protein-coupled receptors mentioned above, there are G protein-coupled adenosine receptors, such as the adenosine A2B receptor (ADORA2B)(48), which is a member of the G protein-coupled adenosine receptor superfamily. ADORA2B and adenosine signal transduction can affect the occurrence and invasion of TNBC, especially in mutant TP53, because the expression of ADORA2B is enhanced by the interaction between the CCAAT box and NF-γ protein (28). In addition, ADORA2B is selectively up-regulated under hypoxic conditions, and hypoxia activates hypoxia factor 1α (HIF-1α) through signal transduction pathways such as Akt and extracellular regulated kinase 1/2 (ERK1/2), and HIF-1α stimulates more adenosine production (49). The study found that cytotoxic cyclic peptide SA-1 can inhibit the expression of ADORA2B (28), to achieve the purpose of inhibiting the occurrence of TNBC. Although GPCRs are almost always located on the cell surface, some GPCRs retain atypical intracellular/nuclear locations and are involved in intracellular signal transduction. These intracellular/nuclear GPCRs, such as kallikrein B1/B2 receptors (B1R/B2R), affect tumor growth, invasion, and angiogenesis. (18), affect tumor growth, invasion, and angiogenesis. For kinin receptors, cell-penetrating kinin receptor antagonists, such as cell-penetrating B1R antagonists (CP-B1Ras) and a cell-penetrating B2R antagonist (CP-B2Ras) (18), are generally used to target B1R and B2R, respectively. For kinin receptors, cell penetrating kinin receptor antagonists, such as cell penetrating B1R antagonists (CP-B1Ras) and cell penetrating B2R antagonists (CP-B2Ras), are generally used to target B1R and B2R, respectively(18). Among them, CP-B1Ras such as SSR240612, NG67, N2000, etc. showed strong anti-cancer activity (29), which could inhibit the proliferation effect caused by the transactivation of EGF receptor and the phosphorylation of MAPK p42/p44 after the activation of B1R. CP-B2Ras can activate the p38kinase/p27kip1 pathway, which in turn arrests the growth of TNBC in the G1 phase and leads to apoptosis (18).

#### 2.1.2 Integrin receptor

The expression of integrin receptors is up-regulated on the surface of tumor cells and is related to the promotion of angiogenesis, cancer cell adhesion, migration, and invasion (31). The common integrin is mainly αVβ3 (50). Studies have found that some peptides can specifically target αVβ3, thereby disrupting integrin-dependent signaling pathways to achieve anti-tumor purposes. A type IV collagen-derived peptide AXT050 specifically binds to αVβ3 to produce an anti-vascular effect unrelated to VEGF inhibits the formation of new blood vessels in TNBC(30), and makes it lack the supply of oxygen and nutrients(51). Another novel peptide ψRGDECHI inhibits the PI3K/Akt pathway involved in integrin-mediated EMT activation after targeting αVβ3 (52), thereby inhibiting the migration and invasion of TNBC cells.

#### 2.1.3 Leptin receptor

Leptin (obesity hormone) is related to obesity-related stimuli, which can be produced by adipose tissue and breast cancer cells (53). Leptin and its receptor (OBR) are overexpressed in TNBC(54). Therefore, the leptin receptor is also a target for TNBC-targeted therapy. The leptin receptor antagonist peptide allo-ACA can specifically bind to OBR, inhibit the expression of downstream signal channels and cyclins induced by OBR activation, and inhibit leptin-stimulated tumor cell proliferation (55). Because leptin is also an angiogenic factor (56), the use of allo-ACA peptides can also achieve anti-angiogenic therapy (32).

#### 2.1.4 Chondroitin sulfate receptor

Chondroitin sulfate proteoglycan 4 (CSPG4) is an antigen receptor with increased expression in tumors, including TNBC. CSPG4 can promote the survival and adhesion of cancer cells and promote tumor growth and metastasis (57). Correspondingly, the polypeptide targeting CSPG4 has an anti-CSPG4 monoclonal antibody (MABS), which can effectively inhibit the proliferation and migration of TNBC by targeting CSPG4 (33).

#### 2.1.5 Wnt receptor

Studies have shown that Wnt receptors are overexpressed in TNBC and induce the proliferation and invasion of TNBC cells through the Wnt/β-catenin signaling pathway(58). RHFZD7 is a recombinant soluble peptide fragment, which can effectively target and antagonize a Wnt receptor Frizzle-7 (FZD7)(34), inhibit the activation of Wnt/β-catenin signaling pathway, and block the initiation of downstream target genes caused by β-catenin entering the nucleus, such as inhibition of c-myc, cyclin D1, survivin, and other target genes can inhibit tumor growth and drug resistance(59), while inhibition of VEGF target genes can inhibit tumor angiogenesis.

#### 2.1.6 Proliferating cell nuclear antigen

Proliferating cell nuclear antigen (PCNA) is essential for DNA replication and repair. During DNA replication, PCNA forms a homotrimer around the DNA chain and loads related proteins and enzymes(60). There are some interdomain connector loop (IDCL) domains in the PCNA monomer, and IDCL contains the motif of the PCNA interacting protein box (PIP-box). The proteins interacting with PCNA regulate DNA replication and repair by binding to these motifs. Therefore, the use of polypeptides to target IDCL to inhibit the interaction between proteins required for DNA replication and repair and PCNA can hinder cell function and promote cell death. The study found a rabbit polyclonal antibody (CAPCNAab) that specifically recognizes the tumor-associated PCNA subtype. CAPCNAab can compete with CAPCNA binding auxiliary proteins and specifically target the CAPCNA in the IDCL of PCNA (35). The replication of DNA in TNBC tumor cells is blocked, and the damaged DNA cannot be repaired and then dies.

### 2.2 Single peptides as decoys target key ligands in the regulatory pathway

In addition to binding to certain receptors on triple-negative breast cancer cells, single peptides can also bind to ligands related to tumor regulation. These peptides can be called peptide decoys. Peptide decoys are molecular traps that bind to certain ligands and play a confusing role, which are the same as receptor proteins. They can be soluble proteins, that is, binding proteins, or inactive cell surface receptors. Soluble peptide decoys have different production mechanisms, including proteolytic cleavage of cell surface receptors, phospholipase C-mediated cleavage, selective splicing of receptor mRNA transcripts, and selective intron polyadenylation (61). These peptide decoys can target key ligands in the breast cancer regulatory pathway, including chemokines, SIN3, etc.

#### 2.2.1 Chemotactic factor

Chemokines are small proteins with low molecular weight and play a key role in tumor immunity and communication between tumor cells and their surrounding environment(62), such as CCR9/CCL25, CXCR5/CXCL13, CXCR4/CXCL12 and CCR7/CCL19 (CCL21), which play a role in cancer growth and metastasis. The decoy peptide can inhibit tumor cells by targeting chemokines, such as the DV1 peptide targeting CXCL12. CXCL12 is a chemokine stromal cell-derived factor-1, and its specific receptor is the chemokine receptor CXCR4. In tumors, CXCR4-CXCL12 is involved in activating a variety of cancer-promoting regulatory mechanisms, thereby promoting tumor proliferation, inhibiting cancer cell apoptosis, and promoting metastasis. DV1 peptide mainly blocks CXCR4 signal transduction by binding to CXCL12 to silence the expression of CXCR4 (36), inhibits Rho-involved RhoA/Rock/Limk signaling pathway (63), and makes Rho protein unable to regulate the tissue, focal adhesion arrangement and intracellular transport of actin stress fibers, thereby inhibiting the migration of tumor cells (64). Of course, silencing the expression of CXCR4 can also inhibit some targets that induce tumor cell migration activity. These targets refer to Akt and its downstream targets activated when stimulating PI3K in CXCR4 signal transduction (36).

#### 2.2.2 SIN3

SIN3 is a key adaptor protein in the co-repressor complex of histone deacetylase (HDAC1/2). As a scaffold connecting DNA-binding transcription factors and chromatin regulators (65), it has a regulatory effect on the proliferation and differentiation of TNBC tumor cells (66). Sin3 includes Sin3A and Sin3B (67), and its structure is composed of four pairs of amphipathic α-helix (PAH1-PAH4) motifs.

The Sin 3-interacting domain (SID) bait is a peptide designed to target the SIN3 complex (PAH2 domain) and induce epigenetic reprogramming in TNBC (68). SID peptide binding to SIN3A can inhibit TGIF1 and then bind to SIN3A, while TGIF1 is a transcription factor involved in the regulation of Wnt signal transduction. TGIF1 can not bind to the PAH2 domain of SIN3A, and then can not promote the expression of the Wnt gene, while the expression of nuclear β-catenin and its target is also inhibited, because the activity of Wnt/β-catenin is not increased, resulting in downstream media such as Axin2, Bcl9 can not be activated(69). It can not further promote the EMT and invasion and metastasis of TNBC tumor cells. The activity was not increased, resulting in downstream mediators such as Axin2 and Bcl9 could not be activated, and it could not further promote the EMT and invasion and metastasis of TNBC tumor cells(70). In addition, some other proteins that can bind to the PAH2 domain, such as the adaptor PF1 (PHF12) and TIEG1 (TGFβ-induced early gene), can not only interact with SIN3A but also interact with SIN3B. First of all, the SID peptide can down-regulate the transcription of genes related to epithelial-mesenchymal transition (EMT) after blocking the interaction between SIN3A and PF1(71). Blocking the interaction between PF1 and SIN3B can inhibit the modification of downstream chromatin of the transcription initiation site by PF1 and restore the effect of RNA polymerase II. SID peptide inhibits the combination of TIEG1 and PAH2, which is to inhibit the TGFβ/SMAD signal transduction pathway involved in TIEG1, so that the expression of Smad2 is down-regulated, and the EMT of tumor is inhibited to a certain extent (72). Of course, the interaction between MAD and SIN3-PAH2 is blocked, which enhances the expression of CDH1 and ESR1 and restores the sensitivity of tumor cells to chemotherapeutic drugs (37).

#### 2.2.3 Transcription factor

Transcription factors are an important part of the signaling pathway. Most of them are proteins. Transcription factors are also related to the progression of tumors by binding to DNA to regulate gene transcription. Targeting transcription factors with inducing peptides can achieve the purpose of treating TNBC.

Among them, STAT3 is one of the many transcription factors, and its overexpression is related to the malignant transformation of tumors including breast cancer. STAT3 has six conserved domains, namely the N-terminal domain followed by the CCD domain, the C-terminal trans-activation domain, the DNA binding domain, the SH2 domain, and the linker domain (38). The activation of STAT3 depends on phosphorylation and dimerization(39). There are several peptides targeting STAT3. The first is a small regulatory peptide ASRPS of STAT3, which mainly regulates the STAT3 vascular endothelial growth factor (VEGF) signaling pathway (38). The combination of ASRPS and the CCD domain of STAT3 can inhibit the phosphorylation of STAT3, thereby reducing the expression of VEGF and inhibiting the formation of tumor blood vessels. The dodecapeptide of the YYVSWPPDMMHY sequence is another peptide targeting STAT3 (39), which mainly prevents the homodimerization of STAT3 and inhibits the stimulation of tumor genes by recognizing and binding to the promoter region of the target gene (73). Studies have shown that YYVSWPPDMMHY peptide down-regulated genes related to angiogenesis (VECGF-A), proliferation-related genes (BIRC5, CDK2, and MCL1) and invasion-related genes (MMP1 and MMP7)(39), which can effectively induce apoptosis and inhibit the growth of TNBC tumor cells.

#### 2.2.4 Anti-apoptotic

Caspase-3 is a protease related to apoptosis and plays an important role in promoting apoptosis. However, in some tumor cells, the function of caspase-3 is inhibited, resulting in the inhibition of apoptosis. For example, after the anti-apoptotic protein-5 (API-5) binds to the acinar protein, the acinar protein cannot be cleaved by caspase-3, and the formation of the active p17 fragment is inhibited, resulting in DNA breakage(74). Therefore, the anti-API5 peptide targeting API-5 promotes the apoptosis of TNBC tumor cells by preventing the API-5/acinar interaction and reactivating the caspase-3 pathway (40).

#### 2.2.5 CIB1

CIB1 is a small cytoplasmic protein, 22kDa (75). It is composed of 10 alpha helices, of which 8 alpha helices form 4 EF-hand domains, and the other 2 alpha helices are binding to the Ca2+ C-terminal domain (76). CIB1 can not only interact with the cytoplasmic tail of integrin αIIb through the hydrophobic pocket hidden by the C-terminal helix 10 to participate in the adhesion and migration of tumor cells(77). CIB1 can also interact with PAK1 to affect the downstream signaling pathways of related signaling pathways such as PI3K/Akt and Ras/RAF/MEK/ERK and regulate the growth and proliferation of TNBC tumor cells (78). Studies have found that CIB1 cyclic peptide inhibitors such as UNC10245131 (41) and UNC10245092 (1), these cyclic peptide inhibitors by targeting CIB1 to prevent its regulation of AKT and ERK carcinogenic pathways, achieve the purpose of intervention TNBC.

### 2.3 Single peptides target TME-related cells

Tumor cells and their environment are a functional whole. Tumor cells and tumor microenvironment (TME) affect each other and jointly promote the occurrence and development of tumors. TME-related cells include immune cells, fibroblasts, and adipocytes (17). These cells are also closely related to tumor growth and metastasis (79). Some single peptides can also play a good role in immune regulation and inhibition of tumor cells by targeting TME-related cells.

#### 2.3.1 Tumor-infiltrating immune cell

Tumor-associated immune cells include tumor-infiltrating lymphocytes, macrophages, neutrophils, etc. Among them, tumor-associated macrophages (TAM) are classically divided into two major groups: M1 and M2. The M1 group is associated with anti-tumor activity, while the M2 group is associated with tumor growth, angiogenesis, migration and invasion, and metastasis, and promotes epithelial-mesenchymal transition (EMT) in TNBC(80). Generally, macrophages are in a balance between M1 and M2 in healthy tissues, while in cancer tissues, the phenotype shifts to M2. Therefore, targeting M2-like macrophages and restoring them to anti-tumor Ml-like macrophages is a direction for cancer treatment. Researchers have found several peptides that can effectively target M2-like macrophages. One is the melittin KLA8-26, which can distinguish M0/M1/M2. When the melittin is attached to the lipid membrane of M2-like macrophages, the membrane will be disturbed, resulting in asymmetry between the two lipid layers and temporary formation of pores, thereby inducing cell death (42). The other is a cyclic peptide with a structure of CSSTRESAC, which can specifically bind to the protein disulfide isomerase A3 (PDIA3) expressed on the cell surface of TAM, namely vitamin D receptor, to achieve the purpose of reducing M2-like macrophages. In addition, there is an “mUNO” peptide, which mainly targets CD206-positive M2-like TAM and converts it into M1-like TAM (43).

In addition to the commonly used method of targeting M2-like macrophages, it can also target other tumor-associated immune cells. C24D peptide is an immunoregulatory therapeutic peptide derived from placental immunoregulatory ferritin targeting CD45 molecule, which consists of 24 amino acids (81). The CD45 receptor is a transmembrane protein tyrosine phosphatase receptor type C, which is present in T cells and NK cells and can inhibit the activation of Src family tyrosine kinases and immune cells in TNBC patients (82). Therefore, after binding to the CD45 receptor on the inhibited leukocytes in TNBC, the C24D peptide can re-activate the Src family of tyrosine kinases, break the inhibition and trigger the intracellular signaling cascade, resulting in an increase in 69+ T cells and 69+ NK cells, inducing CD8+ and activating CD56+ tumor-infiltrating cells to achieve specific killing of TNBC cells (44).

#### 2.3.2 Cancer-associated fibroblasts and adipose stromal cells

Cancer-associated fibroblasts (CAF), also known as tumor mesenchymal stromal cells (MSC), are associated with extracellular matrix (ECM) remodeling, leukocyte recruitment, and immunosuppression in cancer (83), and can induce epithelial-mesenchymal transition (EMT) of cancer cells. Related studies have shown that CAF is heterogeneous and can be derived from different lineages. Some of them are derived from white adipose tissue (WAT) around the tumor, and adipose stromal cells (ASC) are MSC in WAT (84). It is one of the most abundant cell components around the breast tissue, which can cause changes in tumor cell phenotype and promote epithelial-mesenchymal transition and invasiveness of TNBC cells by secreting tumor growth-related hormones and cytokines. Therefore, targeting ASC and depleting it is a new method for the treatment of TNBC. D-CAN peptide, which is made of cyclic peptide WAT7 (sequence CSWKYWFGEC), can induce the apoptosis of ASC and interfere with invasive TNBC (45).

## 3 The combinations of peptides target TNBC to exert immunomodulatory effects

In addition to targeting triple-negative breast cancer in a single form to exert immunomodulatory effects, there are many combinations of peptides, such as vaccines, exosomes, microRNAs, checkpoint inhibitors, etc. These combinations show good results in TNBC treatment (85) **Table 2**.

**Table 2:** The mechanism by which the combinations of peptides target TNBC to exert its immunomodulatory effects.

Catalog		Peptide name	Peptide sequence	Combination modalities	Target	Mechanism	Reference
Breast cancer vaccine		NY-ESO-1 peptide	SLLMWITQV-LSPG-C	NY-ESO-1 vaccine	NY-ESO-1	Activating the response of CD8+ T cells	(86)
		TPIV200 peptide	/	TPIV200 vaccine	TPIV200	Activating the response of CD8+ T cells	(87)
		Human α-lactalbumin peptide	/	Human α-lactalbumin vaccine	Human α-lactalbumin	Activating the response of CD8+ T cells	(88)
Engineered exosomes		c-Met binding peptide	/	Engineered macrophage-derived exosomes		Inhibiting the EMT	(89)
MicroRNA and other immune-related molecular pathways		uPA	/	Elivering anti-miR 21 (pa21) and miR205 (p205)	uPAR	microRNA-21 ↓ microRNA-205 ↑	(90)
				Elivering anti-miRNA-21 or anti-miRNA-10b		microRNA-21 ↓ microRNA-10b ↓	(24)
		cyclic RGD peptide	/	siRNA	TNBC cells	Inhibiting the Wnt/ EMT signal transduction	(91)
		GE11 peptide	YHWYGYTPQNVIGGGGC	siRNA	EGFR	Inhibiting EGFR	(92)
Immune checkpoint inhibitors(ICI)		DPPA-1	NYSKPTDRQYHF	PD-L1	PD1	Inhibiting the interaction between PD1 and PD-L1	(93)
		anti-PD-L1 peptide	CLQKTPKQC	PD-L1	PD-L1	Inhibiting the expression of the PD-1/ PD-L1 interaction axis	(94)
		PEP	NYSKPTDRQYHF	PD-L1	PD-L1	Inhibiting the circulation of PD-L1	(95)
Adoptive cell therapy-chimeric antigen receptor T cells		NCL peptide	KMAPPPKEV; VLSNLSYSA	NCL-specific T cells	NCL	Secific killing of NCL overexpressed tumor cells	(94)
		PLAC1 peptide	VLCSIDWFM	PLAC1-specific TCR engineered T cell	PLAC 1	Specific killing of PLAC 1 overexpressed tumor cells	(96)
Drug-peptide conjugates	Cell penetrating peptide	CPP	RLYMRYYSPTTRRYG	Elivering DOX	TNBC cells	Causing a short or long-term stable imbalance of the membrane at the binding site of the two, and CPP can flow into tumor cells.	(97)
				ZER-HPβCD			98)
				Elivering Rictor siRNA			(99)
Tumor penetrating peptide		iRG	/	Elivering DOX	TNBC cells	Making anticancer drugs enter tumor cells to enhance the therapeutic effect	(100)
Other peptides		TH19P01	Ac-­GVRAKAGVRN(Nle)FKSESY	Elivering DOX	SORT1	Selective treatment of TNBC cells expressing SORT1	(101)
		GE 11	YHWYGYTPQNVI	Elivering DOX	EGFR	Selective treatment of TNBC cells expressing EGFR	(102)
		18-4 peptide	/	Elivering DOX	K1	Selective treatment of TNBC cells expressing K1	(11)
		EBP	CAHKHVHHVPVRL	Elivering DOX	Endothelial glycoprotein	Selective treatment of TNBC cells expressing endothelial glycoprotein	(103)
		BP 10 peptide	CPWKRMEKKRSHL	Elivering DOX	PROCR	Selective treatment of TNBC cells expressing PROCR	(104)
		AXT050 peptide	/	Elivering DOX	Integrins	Selective treatment of TNBC cells expressing integrins	(30)
		tLyP-1peptide	CGNKRTR	Elivering DOX and paclitaxel	The scavenger receptor type BI and NRP-1	Selective treatment of TNBC cells expressing the scavenger receptor type BI and NRP-1	(27; 105)
		iNGRt peptide	CRNGR	Elivering DTX	NRP-1	Selective treatment of TNBC cells expressing NRP-1	(106)

/, The structure of the peptide is not mentioned in the current reference, and it is not clear.

### 3.1 Breast cancer vaccine

Cancer vaccines mainly recognize tumor-associated antigens by stimulating the immune system to produce corresponding antibodies to achieve the effect of prevention and treatment. In TNBC, many new antigens have been identified, such as a cancer-testis antigen, folate receptor α (Frα), human α-lactalbumin, and so on (87). However, many of these antigens are short peptides with poor immunogenicity and cannot stimulate the cellular immune response well. Therefore, adjuvants are generally added to these antigenic peptides to enhance the immune response and improve the efficacy of the vaccine. Common adjuvants are MPL or MF (107). NY-ESO-1, the first cancer-testis antigen, is a highly immunogenic antigen target present in triple-negative breast cancer. Studies have developed the NY-ESO-1 vaccine using cowpea mosaic virus (CPMV) as an adjuvant (86). CPMV has strong immune stimulation due to its protein structure and capsized nucleic acid, which enhances the uptake of NY-ESO-1 peptide by antigen-presenting cells and the subsequent response of CD8+ T cells. Secondly, folate receptor α (Frα) TPIV200 is also a candidate vaccine and is being studied (87). Human α-lactalbumin is an antigen that is only expressed in the lactating mammary gland of normal tissues, but is also expressed in TNBC, and has the potential to be a candidate vaccine target (88). The combination of antigen peptides and adjuvants to make vaccines stimulate the body to strengthen the humoral immune response and a cellular immune response is believed to play a greater role in the prevention and treatment of triple-negative breast cancer in the future.

### 3.2 Engineered exosomes

Exosomes are extracellular vesicles that can be found in almost all cells and affect the growth and metastasis of tumor cells by participating in many cell signal transduction processes. They have now been used to deliver some important molecules (87). It has been reported that when the antigen peptide in cancer is carried by exosomes, the body will produce stronger immune stimulation on it, so exosomes are also tried to be used as immunogen carriers(108). Studies have produced engineered macrophage-derived exosomes for carrying c-Met binding peptides. C-Met is a mesenchymal-epithelial transforming factor, and its abnormal signals will lead to poor prognosis and increased metastasis of triple-negative breast cancer (89). Using c-Met binding peptides combined with exosomes can enhance the immune response and inhibit the expression of c-Met, thereby achieving therapeutic effects.

### 3.3 MicroRNA and other immune-related molecular pathways

The growth, metastasis, and recurrence of TNBC are also closely related to the imbalance of immune-related molecules such as microRNAs (miRNA) and short interfering RNA (siRNA) (87). Regulating the expression of these molecules by targeting the delivery of miRNA or siRNA can well inhibit breast cancer cells.

MiRNAs are generally small non-coding RNA molecules composed of about 20 nucleotides. MiRNAs can bind to the complementary sequence bases of endogenous mRNAs and regulate gene expression by interacting with the 3’ untranslated region of the target gene, thereby interfering with the transcription and translation of specific genes (109). In TNBC, the disordered expression of miRNAs can lead to the growth, metastasis, and recurrence of tumor cells. Among them, the overexpression of microRNA-21, miRNA-10b, and miRNA-21 and the down-regulation of microRNA-205 often occur (24, 90). In the corresponding study, plasmid anti-miR21 (pa21) and plasmid miR205 (p205) were coated in urokinase plasminogen activator peptide (uPA). UPA can target urokinase plasminogen activator receptor (uPAR) in the TNBC cell membrane, thereby delivering anti-miR 21 (pa21) and miR205 (p205) to tumor cells, playing a role in simultaneously down-regulating microRNA-21 levels and up-regulating microRNA-205 levels (90). At the same time, there are also studies using uPA to target the delivery of anti-miRNA-21 or anti-miRNA-10b, antagonizing the accumulation of multiple endogenous miRNAs to achieve the purpose of treatment (24).

Immune-related molecules In addition to the aforementioned miRNAs, there are short interfering RNAs (siRNAs). The siRNA molecule is a double-stranded short-chain oligonucleotide that can specifically match the sequence of the mRNA molecule, thereby causing specific gene silencing, which is of great significance for the treatment of cancer(110). The experimental cyclic RGD peptide delivery therapeutic siRNA can down-regulate PRC 2-mediated H3 K27 trimethylation and Wnt/EMT signal transduction, which changes the phosphorylation spectrum of several kinases in TNBC cells and affects the carcinogenic relationship (91). In addition to the use of cyclic RGD peptides to deliver siRNA, GE11 peptide is also used, which is an anti-EGFR peptide that can actively bind to TNBC cells overexpressing epidermal growth factor receptor (EGFR), which is beneficial for siRNA to better target these tumor cells (92).

### 3.4 Immune checkpoint inhibitors

Immune checkpoints determine the activation or inhibition of the host immune response in anti-tumor immunity. Common immune checkpoints are PD-L1 and PD-L2. Tumor cells evade immune surveillance of T cells by up-regulating programmed death ligand (PD-L1) on the cell surface (111). Moreover, studies have shown that the expression of PD-L1 is much higher in TNBC cells than in other subtypes of breast cancer(112). Therefore, the use of immune checkpoint inhibitors (ICI) can restore the host’s immune response and enhance the clearance of tumor cells. At the same time, some studies have found that some peptides can act as immune checkpoint inhibitors and have good anti-tumor effects. The general method used to block immune checkpoints is to block the interaction between programmed cell death protein 1 (PD1) and PD-L1, such as a D-peptide antagonist (DPPA-1), DPPA-1 is the hydrolysis release product LAG-DPPA-1 of the sequence MMP-2 on the CD peptide (93). In addition, the anti-PD-L1 peptide can be used to directly target PD-L1, activate autophagy by inhibiting PD-L1, and restore T cell-mediated killing in TNBC tumors (94). Although blocking PD-L1 does prevent the expression of the PD-1/PD-L1 interaction axis to a certain extent, it generally shows a good therapeutic effect. In some patients, PD-L1 is circulating in tumor cells, and then PD-L1 is re-expressed on the cell surface (113). Therefore, blocking the circulation of PD-L1 is the key. Some studies have proposed the use of PD-L1 targeting D peptide (NYSKPTDRQYHF, PEP). After PEP binds to PD-L1 on the surface of cancer cells, it will guide PD-L1 into lysosomes, making PD-L1 degraded and unable to circulate again, thereby down-regulating the expression of PD-L1 and restoring the killing effect of T cells on TNBC tumor cells (95).

### 3.5 Adoptive cell therapy-chimeric antigen receptor T cells

Adoptive cell therapy (ACT) is a highly effective and promising treatment method, which is generally activated by tumor-specific lymphocytes including TIL, CD8 + cells and CD4 + helper cells, or other cells of the immune system such as NK cells and DC cells, and then infused into patients to kill tumor cells. Chimeric antigen receptor T cell therapy is a kind of adoptive cell therapy. These T cells can specifically recognize tumor-specific antigen (TSA) or tumor-associated antigen (TAA), especially TSA, through specific T cell receptors (TCR) to achieve tumor-targeted therapy (87).

One of the chimeric antigen receptor T cells mentioned first is nucleolin (NCL) specific T cells. NCL is a protein that exists in the nucleolus, cytoplasm, and cell surface of eukaryotic cells, and has a variety of biological functions because NCL is overexpressed in TNBC and is associated with metastasis and poor prognosis of TNBC cells (114). Therefore, NCL has become a potential target for TNBC treatment. Researchers have engineered NCL-specific T cells and found that they have a good specific killing effect on NCL-overexpressing tumor cells (94). In addition, there is also a PLAC1-specific TCR-engineered T cell. Placenta-specific antigen 1 (PLAC1) is one of the cancer-testis antigens (CTA), which is expressed in malignant tumors such as breast cancer and is related to the proliferation and migration of tumor cells(115). Effective engineering of CD8 + T cells to express TCRs that recognize human leukocyte antigen (HLA) -restricted PLAC1 peptides can specifically kill tumor cells overexpressing PLAC1. Although PLAC1 is also expressed in normal tissues of the testis and placenta, it is not affected by TCR-engineered T cells because germ cells do not express MHC molecules and do not present antigens(116).

### 3.6 Drug-peptide conjugates

Drug-peptide conjugates (PDC) are a unique class of drug carriers that couple small molecule drugs with peptides. These peptides can modify and deliver the corresponding drugs to tumor cells, enhance the intracellular transport of drugs, and improve the lethality of the corresponding drugs (117). There are many kinds of peptides applied in PDC, among which cell-penetrating peptide (CPP) and tumor-penetrating peptide (TTP) are the most widely used. At the same time, some other peptides are being studied, which are mainly modified by small-sized drugs such as oligonucleotides and chemotherapeutic drugs.

#### 3.6.1 Cell-penetrating peptide

CPP is composed of natural amino acids with high biocompatibility and effective tissue penetration. The positively charged amino acids on CPP and the negatively charged membrane phospholipid bilayer on the surface of tumor cells form an electrostatic interaction. At the same time, the hydrophobic amino acids of CPP interact with the hydrophobic core of the tumor cell membrane, which makes the lipid bilayer sparse, causing a short or long-term stable imbalance of the membrane at the binding site of the two, and CPP can flow into tumor cells (117).

Because the lack of targeting of chemotherapeutic drugs often damages normal tissue cells to produce toxic effects, the use of CPP-modified chemotherapeutic drugs has become a research hotspot. Some studies have modified the chemotherapeutic drug doxorubicin (DOX) with tumor-homing CPP (RLYMRYYSPTTRRYG) and loaded it into nanoparticles (NP) and found that it has good blood compatibility and biocompatibility. The effect of inhibiting the growth of TNBC cells is more significant than that of DOX alone (97). In addition to some commonly used chemotherapeutic drugs, some natural anticancer drugs have gradually attracted people’s attention. Here, the anticancer drug Zerumbone (ZER, M.218.34g/mol) derived from the rhizome of wild ginger Zingiber Zerumbet Smith is mentioned. It is a sesquiterpene compound that can increase the activation of Bax to induce apoptosis of breast cancer cells and inhibit the proliferation of tumor cells by inhibiting the expression of the Ki-67 protein (118). ZER achieves targeted therapy of ZER by using CPP-modified ZER (ZER-HPβCD) encapsulated in hydroxypropyl-β-cyclodextrin (98). In addition, oligonucleotides have always been a good choice for the treatment of cancer, such as small interfering RNA (siRNA), which can effectively target the table of silent genes (119). However, due to the relatively unstable physiological environment of naked siRNA in the blood system, digestive system, and intracellular lysosomes, it is easy to be removed when used alone, so the CPP delivery method shows great potential. In some experiments, CPP-modified GO nanoparticles loaded with Rictor siRNA were injected into nude mice, which significantly inhibited the growth of TNBC tumors. This may be because Rictor siRNA further inhibited the phosphorylation of Akt and p70s6k by inhibiting the expression of Rictor, as well as the subsequent PI3K/Akt/mTOR signal transduction (99).

#### 3.6.2 Tumor penetrating peptide

TTP is mainly targeted at tumor cells, and drug delivery destroys cancer cells through different mechanisms. IRG is a commonly used cyclic tumor penetrating peptide, which is composed of a disulfide-linked RGD module and an overlapping C-terminal R module (120). The RGD module can bind to the highly expressed αvβ3 or αvβ5 integrin on TNBC tumor cells, making anticancer drugs enter tumor cells to enhance the therapeutic effect (121). Studies have confirmed that RGD-modified RBC membrane and then encapsulating DOX can ensure its good biological stability and low cytotoxicity *in vivo* (99).

#### 3.6.3 Other peptides

PDC has a class of antibody-drug conjugates (ADC), which recognize TAA or TSA through corresponding antibodies. These peptides as antibodies do not have anti-cancer effects themselves, but after binding to tumor cells, they can deliver the corresponding drugs and kill the target cancer cells by internalizing the cells (87). There are many potential peptides in ADC. The following will list some peptides that are being studied in recent years.

The first thing to be mentioned is the newly developed peptide TH19P01 targeting neurotensin receptor 3 (SORT1). SORT1 belongs to the receptor of the VPS10P family and is abnormally expressed in human cancers such as breast cancer, ovarian cancer, and pancreatic cancer. TH19P01 can be combined with many anticancer drugs such as DOX, and then selectively treat TNBC cells containing SORT1 (101). Epidermal growth factor receptor (EGFR) has become a promising target because it is overexpressed in TNBC cancer cells. A twelve peptide named GE 11 (YHWYGYTPQNVI) can effectively bind EGFR and release DOX to kill tumor cells (102). In addition, keratin 1 (K1) is a novel receptor that is highly expressed on breast cancer cells and can also be used as a target for drug delivery. Peptide 18-4 was designed and then conjugated with a DOX-containing acid-sensitive hydrazone linker to achieve targeted killing of cancer cells(11). In addition, endothelial glycoprotein binding peptide (EBP) can also be used to modify DOX and target TNBC cells(103). Similarly, peptide BP 10 has a strong affinity for the new cancer stem cell marker protein C receptor (PROCR) in tumor tissues of TNBC patients. By targeting TNBC cells expressing PROCR, DOX is released and the treatment efficiency is improved (104). The AXT 050 peptide is a collagen-IV-derived peptide that can target tumor-associated integrins and increase the accumulation of drugs in tumor cells (30). Here we also mention a tumor-homing peptide tLyP-1 peptide that can load DOX and paclitaxel and target tumor cells by binding to the overexpressed scavenger receptor type B I and neuropilin receptor 1 (NRP-1) in TNBC cells(27, 91, 105). The iNGRt peptide can also target NRP-1, which enhances anticancer activity by attaching to the surface of NP loaded with the anticancer drug docetaxel (DTX) (106).

## 4 Conclusion

TNBC is a highly heterogeneous subtype of breast cancer, and its incidence is increasing year by year. Due to the lack of effective therapeutic targets, it has a higher recurrence and metastasis rate, and mortality rate than ordinary breast cancer. Surgery plus chemotherapy is the main method of clinical treatment of TNBC, but the curative effect is very little (17). So far, the treatment progress of TNBC has lagged far behind other breast cancer subtypes, and the treatment of TNBC is still a huge challenge. With the continuous improvement of the understanding of the immunogenicity of TNBC, TNBC immunotherapy has developed rapidly in recent years and has shown strong therapeutic potential (122). However, there are still some challenges in the immunotherapy of TNBC. For example, some patients have poor responses to immunotherapy, and highly accurate prediction methods are needed to screen out responders for treatment and further explore the reasons for poor responses. Many TNBC-associated antigens are not only highly expressed in tumor cells, but also expressed in normal cells to varying degrees. Immunotherapy targeting these antigens may damage normal tissues and cause adverse reactions(123). TNBC cells have strong migration ability and poor response to targeted drug treatment. Therefore, it is necessary to further find new TNBC-specific therapeutic targets and promote personalized treatment in the direction of future development.

In the future, the combination of targeted therapy and immunotherapy is also expected to improve the efficacy of anti-tumor therapy. The combination of the two has a synergistic therapeutic effect, which may be better than the simple superposition of the effects of a single component (124). Targeted therapy can accurately overcome various forms of immunosuppression in the tumor microenvironment and promote anti-tumor immune regulation. The combination of targeted therapy and immunotherapy includes a combination of various immunotherapy drugs and new treatments such as standard therapy and targeted therapy (125). Among these new treatments, peptides have become a new star in anti-tumor therapy because of their advantages such as small molecular weight, high biocompatibility, and easy synthesis, and numerous researchers have been competing to study them. Of course, peptides also have some shortcomings, such as being unstable and easily decomposed *in vivo*, having short half-life, and most cannot be taken orally(123). For various reasons, the research of many peptides as drugs has been stuck in the theoretical stage, failing to carry out clinical trials and further promotion, but it is believed that there will be a breakthrough shortly.

At the same time, with the development of sequencing technology and medical big data processing, it has become a hot spot in current medical development to adopt accurate medical strategies to deal with highly heterogeneous diseases such as tumors and to select reasonable combination therapy according to the tumor characteristics of each patient (126). Using whole exome sequencing, targeted sequencing, and transcriptome sequencing, we can analyze the tumor-specific cancer driver genes and the abnormal signaling pathways involved in each patient’s tumor, such as PI3K/AKT, RAS/MAPK, DNA damage repair, cell cycle regulation, and transcriptional regulation (127). Most of the drugs targeting these pathways are currently in clinical trials, and the treatment strategies combined with other therapies, including anti-PD-1/PDL1 antibodies, have attracted much attention. In the future, clinical trials with larger sample sizes will be carried out to explore their effects on survival and disease development (17). It is believed that through further research, the treatment of TNBC will achieve greater breakthroughs in the future.

## Author contributions

YH: conceptualization, writing – original draft, writing – review & editing. LS: funding acquisition, writing – review & editing. AZ: conceptualization, visualization.

## Glossary

**Table:**

TNBC	triple-negative breast cancer
ER	estrogen receptor
PR	progesterone receptor
HER2	human epidermal growth factor receptor 2
PCR	pathological complete response
ICI	immune checkpoint inhibitors
GPCRs	G protein-coupled receptors
PAR1	protease-activated receptor 1
OBR	obesity hormone receptor
CSPG4	chondroitin sulfate proteoglycan 4
FZD7	Frizzle-7
PCNA	proliferating cell nuclear antigen
IDCL	interdomain connector loop
PIP-box	PCNA interacting protein box
SID	Sin 3-interacting domain
VEGF	vascular endothelial growth factor
API-5	anti-apoptotic protein-5
TME	tumor microenvironment
TAM	tumor-associated macrophages
EMT	epithelial-mesenchymal transition
CAF	cancer-associated fibroblasts
MSC	mesenchymal stromal cells
WAT	white adipose tissue
ASC	adipose stromal cells
Frα	folate receptor α
CPMV	cowpea mosaic virus
miRNA	microRNAs
siRNA	short interfering RNA
pa 21	plasmid anti-miR21
p205	plasmid miR205
uPA	urokinase plasminogen activator peptide
uPAR	urokinase plasminogen activator receptor
pa 21	anti-miR 21
p205	miR205
EGFR	epidermal growth factor receptor
PD-L1	programmed death ligand
PD1	protein 1
ACT	adoptive cell therapy
TSA	tumor-specific antigen
TAA	tumor-associated antigen
TCR	T cell receptor
NCL	nucleolin
PLAC1	placenta-specific antigen 1
CTA	cancer-testis antigens
HLA	human leukocyte antigen
PDC	drug-peptide conjugates
CPP	cell penetrating peptide
TTP	tumor penetrating peptide
DOX	doxorubicin
NP	nanoparticles
ADC	antibody drug conjugates
SORT1	neurotensin receptor 3
K1	keratin 1
PROCR	protein C receptor
DTX	docetaxel.
